# Linking functional connectivity and dynamic properties of resting-state networks

**DOI:** 10.1038/s41598-017-16789-1

**Published:** 2017-11-30

**Authors:** Won Hee Lee, Sophia Frangou

**Affiliations:** Department of Psychiatry, Icahn School of Medicine at Mount Sinai, New York, NY 10029 USA

## Abstract

Spontaneous brain activity is organized into resting-state networks (RSNs) involved in internally-guided, higher-order mental functions (default mode, central executive and salience networks) and externally-driven, specialized sensory and motor processing (auditory, visual and sensorimotor networks). RSNs are characterized by their functional connectivity in terms of within-network cohesion and between-network integration, and by their dynamic properties in terms of synchrony and metastability. We examined the relationship between functional connectivity and dynamic network features using fMRI data and an anatomically constrained Kuramoto model. Extrapolating from simulated data, synchrony and metastability across the RSNs emerged at coupling strengths of 5 ≤ *k* ≤ 12. In the empirical RSNs, higher metastability and synchrony were respectively associated with greater cohesion and lower integration. Consistent with their dual role in supporting both sustained and diverse mental operations, higher-order RSNs had lower metastability and synchrony. Sensory and motor RSNs showed greater cohesion and metastability, likely to respectively reflect their functional specialization and their greater capacity for altering network states in response to multiple and diverse external demands. Our findings suggest that functional and dynamic RSN properties are closely linked and expand our understanding of the neural architectures that support optimal brain function.

## Introduction

Brain activity at rest is organized into functional resting-state networks (RSNs) defined by their spatiotemporal configuration and functional roles^1–4^. The default mode network (DMN)^5^, the central executive network (CEN)^6^ and the salience network (SAL)^7^ support diverse and mostly internally-guided processes. The DMN is implicated in self-referential and integrative processes^5^, the CEN is involved in goal-directed selection of stimuli and responses^6^, and the SAL processes information relating to varied forms of cognitive, affective and homeostatic salience^7^. Further networks, particularly the auditory (AN), visual (VN) and sensorimotor (SMN) networks, are known to support more specialized and mostly externally-driven functions^8^.

Using functional connectivity metrics, it is possible to characterize RSNs based on their internal cohesion and integration. RSNs with high within-network connectivity can be considered cohesive while those with low within-network connectivity can be considered incohesive^9^. Similarly, RSNs with high between-network connectivity can be designated as connector networks while those with low between-network connectivity can be designated as provincial networks^9^. In the adult brain, the DMN has a unique role as a cohesive connector, the CEN and SAL behave as incohesive connectors while the AN, VN and SMN function as cohesive provincial networks^9^. These functional properties are considered essential components of the optimal functional configuration of the human brain.

More recently, the functional behavior of brain networks has been considered in terms of their synchrony and metastability that attempt to capture dynamic changes in network configuration. Synchrony is a measure of the synchronization of the individual network regions while metastability refers to the degree of variability in network states over time^10,11^. Optimal brain function is thought to occur at levels of synchrony and metastability that allow both efficient information flow and the adaptive changes to network states in response to external or internal demands^10,12–18^. Previous studies have found that synchrony and metastability differ between RSNs^12,13,19^. However, it is unclear whether such differences are associated with the functional connectivity profiles of the RSNs. The aim of the current paper is to test whether the functional and dynamic properties of RSNs are indeed linked. Specifically, we hypothesized that the role of internally-guided RSNs as connector networks would be associated with lower metastability and synchrony; lower metastability would enable connector networks to maintain relatively stable configurations over time to sustain ongoing mental operations, consistent with the dynamic behavior of these networks during tasks^13^, while lower synchrony at rest would enable connector networks to adopt variable configurations in order to support diverse mental operations^13^. In contrast, we also hypothesized that the specialized and externally-driven function of provincial RSNs would be associated with greater synchrony and metastability that will enable them to change their dynamic states in response to variable and competing inputs while maintaining internal network cohesion consistent with their more circumscribed functional role.

## Results

**Figure 1:**
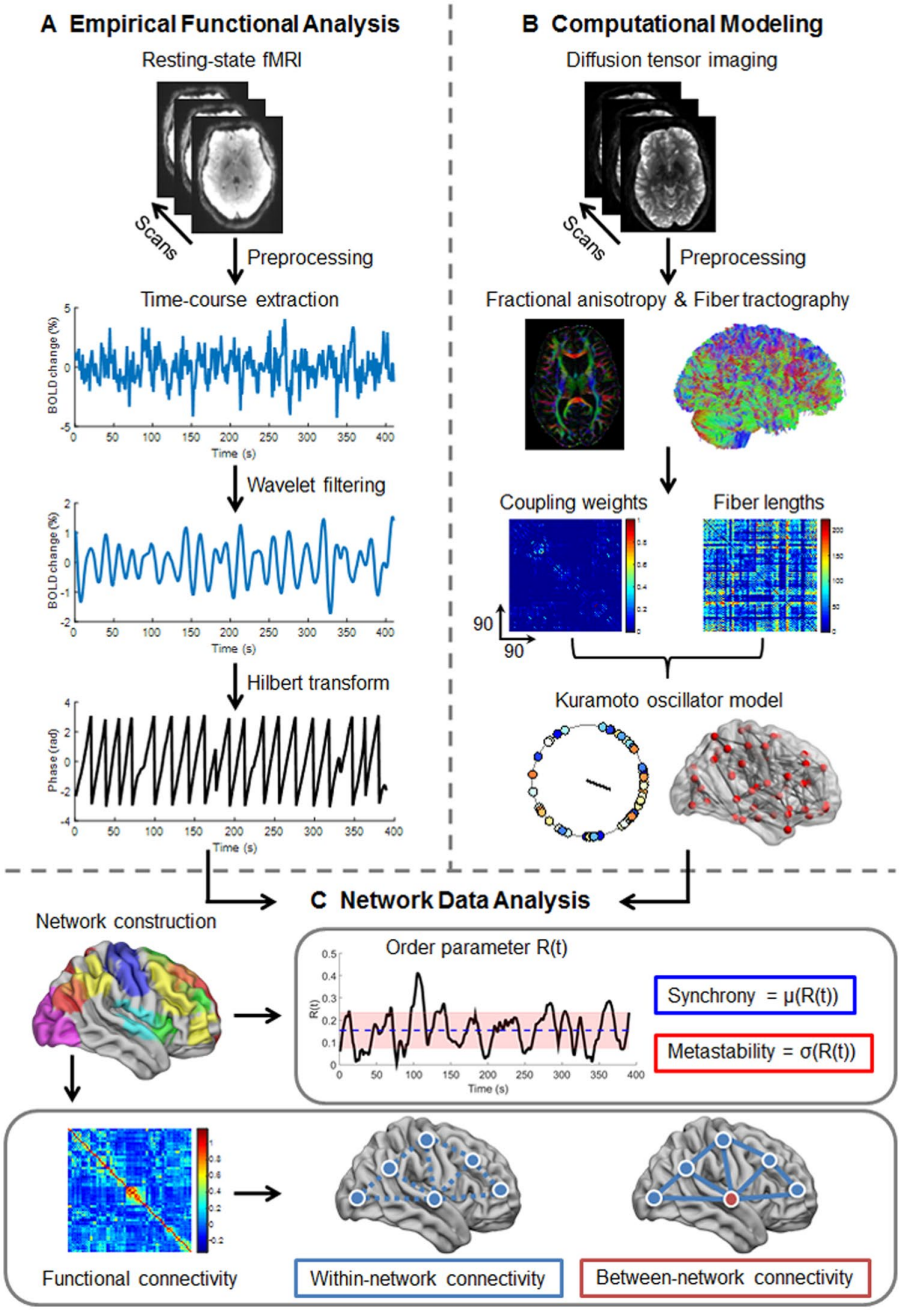
Overview of the workflow. (**A**) Resting-state fMRI and diffusion tensor imaging (DTI) were obtained from healthy participants. After fMRI image preprocessing, 90 cortical and subcortical regions (45 in each hemisphere) were defined using the Automated Anatomical Labeling (AAL) atlas. A wavelet decomposition technique was applied to the regional fMRI time series to extract frequency-band specific fMRI signals (scale 4; 0.03–0.06 Hz). Hilbert transformation was used to acquire phase representation of empirical fMRI time series. (**B**) Neural dynamics were simulated using the Kuramoto oscillator model constrained by empirical structural connectivity (coupling weights and fiber lengths) derived from the DTI data. (**C**) Six major resting-state networks (RSNs) were extracted (default mode network, central executive network, salience network, sensorimotor network, visual network, and auditory network). Measures of synchrony and metastability were computed using the order parameter $R(t)$ as the mean (blue) and standard deviation (red) of the order parameter amplitude across time, respectively, for both empirical and simulated phase time courses. The dynamic properties of the empirical and simulated data of each RSN were compared. In parallel, the empirical within- and between-network functional connectivity of each RSN were computed and their relationship with the dynamic properties of the empirical RSNs was examined.

An overview of the study workflow is shown in Fig. 1. Resting-state functional magnetic imaging (rs-fMRI) and diffusion tensor imaging (DTI) data were obtained from 30 healthy participants. Six RSNs were extracted (DMN, CEN, SAL, SMN, VN, and AN). For each empirical RSN, we computed within- and between-network connectivity, synchrony and metastability. In parallel, we used a Kuramoto oscillator model constrained by DTI-derived structural connectivity to compute measures of synchrony and metastability for simulated RSNs. For each RSN, we compared the dynamic properties of empirical and simulated data and examined the relationship between functional connectivity and dynamic metrics.

### Empirical connectivity, synchrony and metastability of the RSNs

**Figure 2:**
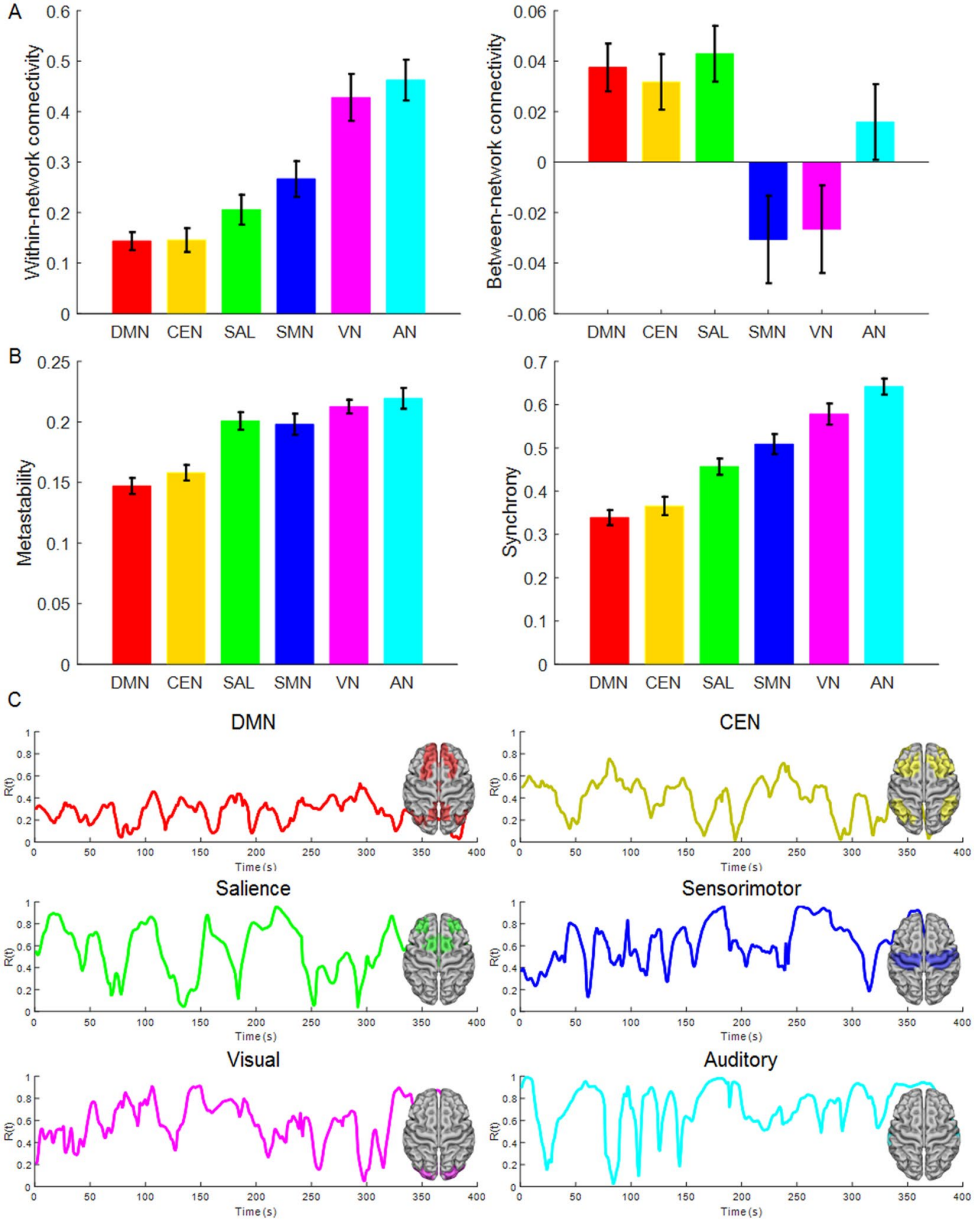
Empirical network connectivity, synchrony and metastability. (**A**) The mean and standard error of mean (SEM) of empirical within- and between-network connectivity for each resting-state network (RSN) across all participants. (**B**) The mean and SEM of empirical metastability and synchrony for each RSN across all participants. (**C**) Temporal fluctuations of the order parameter amplitude $R(t)$ of the functional magnetic resonance imaging (fMRI) signal in each RSN from a single representative subject. DMN = default mode network, CEN = central executive network, SAL = salience network, SMN = sensorimotor network, VN = visual network, AN = auditory network.

The within- and between-network functional connectivity of each RSN, respectively reflecting cohesion and integration, are shown in Fig. 2A. The metastability and synchrony of each RSN are shown in Fig. 2B. The temporal fluctuation of the order parameter amplitude $R(t)$ for each empirical RSN from a single representative subject is shown in Fig. 2C. The metastability, synchrony and functional connectivity of all RSNs were normally distributed (all Kolmogorov–Smirnov-derived *p* > 0.1). None of these measures was significantly influenced by age, sex and IQ based on a series of multiple regression analyses with bootstrapping (all *p* > 0.12; for details see Supplementary Table S3).

Overall, externally-driven provincial networks (AV, VN and SMN) had numerically higher synchrony and metastability than internally-guided connector networks (DMN and CEN). The variance of metastability and synchrony was not statistically different between networks despite differences in network size (all *p* > 0.09 based on Levene’s test for equality to variance). Analyses of variance with bootstrapping (n = 1000) showed that the networks differed in metastability (F_5_ = 64.08; *p* < 0.0001; 95% confidence interval 0.18, 0.19) and synchrony (F_5_ = 125.44; *p* < 0.0001; 95% confidence interval 0.47, 0.49). The results remained significant after co-varying for network size (number of component regions), age, sex and IQ. Bonferroni corrected post-hoc pairwise comparisons showed that metastability was comparable between connector networks (DMN and CEN; *p* = 0.56) and between provincial networks (SMN, VN, AN; *p* > 0.09). However, connector networks (DMN, CEN) had significantly lower metastability than all other networks (all *p* < 0.001). Bonferroni corrected post-hoc pairwise comparisons showed that synchrony was comparable between connector networks (DMN and CEN; *p* = 0.98) but all other pairwise comparisons were significant (all *p* < 0.0001).

**Figure 3:**
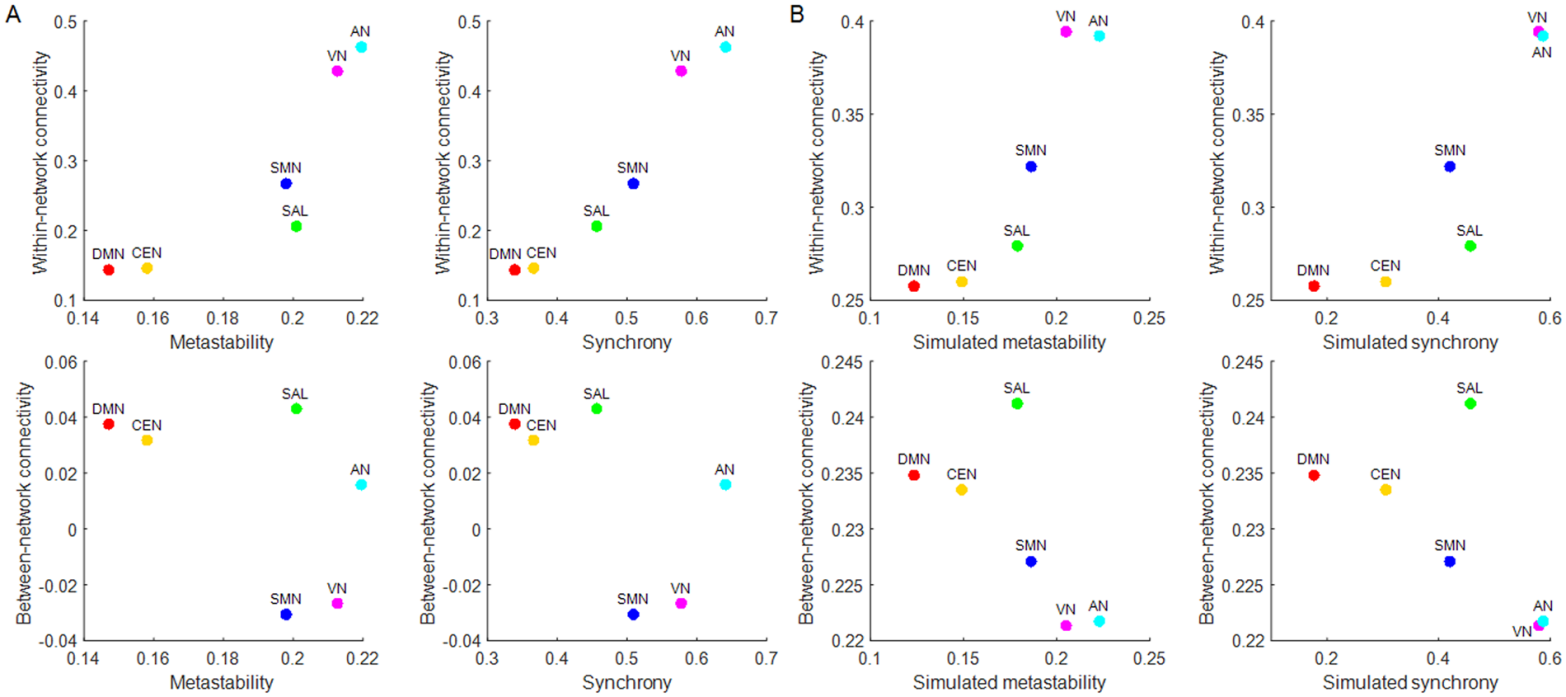
Relationship between functional connectivity and dynamic network properties. Cartographic representation of the cohesion (correlation-based, within-network connectivity), integration (correlation-based, between-network connectivity) of the empirical resting-state networks with (**A**) empirical metastability and synchrony and with (**B**) simulated metastability and synchrony at optimal coupling strength (*k*), equal to the maximal metastability of each RSN. Each network is represented in a position defined by its average values for these measures. DMN = default mode network, CEN = central executive network, SAL = salience network, SMN = sensorimotor network, VN = visual network, AN = auditory network.

Correlation analyses with bootstrapping (n = 1000) revealed that network metastability was positively associated with network cohesion (within-network connectivity) (*r* = 0.69, *p* < 0.0001, 95% confidence interval 0.61, 0.76) and negatively associated with network integration (between-network connectivity) (*r* = −0.17, *p* = 0.02, 95% confidence interval −0.30, −0.05). Similar analyses showed that network synchrony was positively associated with network cohesion (*r* = 0.93, *p* < 0.0001, 95% confidence interval 0.92, 0.95) and negatively associated with network integration (*r* = −0.20, *p* = 0.005, 95% confidence interval −0.32, −0.09). The relationship between the functional and dynamic properties of the empirical RSNs is visualized in Fig. 3A.

### Simulated metastability and synchrony as a function of coupling strength

**Figure 4:**
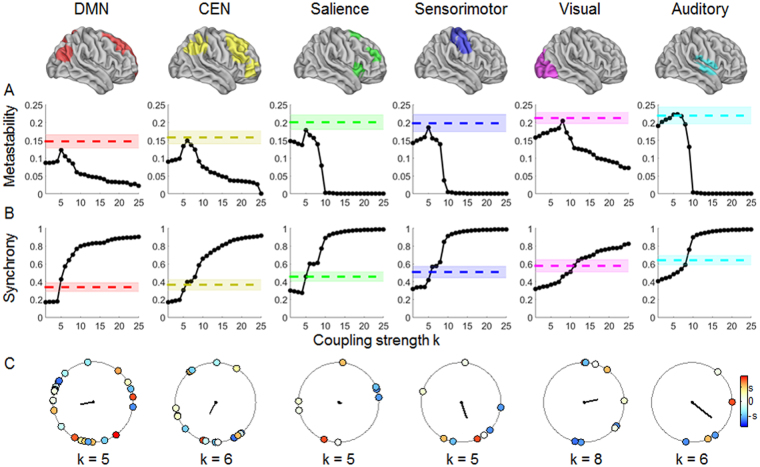
Simulated metastability and synchrony as a function of coupling strength. (**A**) Metastability (black line) and (**B**) synchrony (black line) generated using the Kuramoto oscillator model as a function of structural coupling strength *k* and comparison with empirical metastability and synchrony for each resting-state network (RSN). Color-coded dashed lines and shaded bands indicate, respectively, the mean and standard deviation of the empirical data across all participants. (**C**) Color-coded phase circle diagram at t = 390 s for coupling strengths at which the simulated metastability is maximized. Solid lines in black represent the amplitude of the order parameter, R(390 s) = 0.3, 0.3, 0.1, 0.4, 0.4, and 0.6, respectively corresponding to *k* = 5, 6, 5, 5, 8, and 6. The color of each oscillator represents the natural frequency deviation from the mean (in the color bar, S is the standard deviation of the natural frequencies). DMN = default mode network, CEN = central executive network, SAL = salience network, SMN = sensorimotor network, VN = visual network, AN = auditory network.

The simulated metastability and synchrony of each RSN as a function of structural coupling strength is depicted in Fig. 4A,B while Fig. 4C shows a snapshot of phase relationships of the Kuramoto model at t = 390 s for each RSN at the coupling strength of maximal metastability. The goodness-of-fit between the computational model and the empirical data for a range of coupling strengths was provided in terms of the absolute difference in metastability and synchrony. The lowest absolute difference between the empirical and simulated data for each RSN, ranging from 0.3 to 2% for metastability and from 0.02 to 0.9% for synchrony, was observed at the point of maximal metastability derived from the Kuramoto model. The maximal simulated metastability was observed at *k* = 5 in the DMN, SAL, and SMN, at *k* = 6 in the CEN and AN, and at *k* = 8 in the VN. These results suggest that varying the coupling strength *k* allowed us to reproduce the dynamic properties (metastability and synchrony) of the empirical RSNs. We examined the relationship between simulated metastability and synchrony versus empirical within- and between-network connectivity. We compared the empirical data to the simulated data at *k* equal to the maximal metastability of each RSN. As shown in Fig. 3B, the empirical and simulated metastability and synchrony of each RSN yielded comparable results with regards to their relationship to RSN cohesion and integration. These results demonstrate the computational model reproduced the dynamic properties of the empirical RSNs and their relationship with functional connectivity characteristics (within- and between-network connectivity).

## Discussion

We examined the relationship between the functional and dynamic properties of the DMN, CEN and SAL, which represent internally-guided, connector networks, and the AN, VN and SMN, which represent externally-driven provincial networks concerned with processing specialized functions. Across RSNs, metastability and synchrony were positively correlated with within-network connectivity and negatively correlated with between-network connectivity, indicating that increased metastability and synchrony were associated with elevated cohesion and decreased functional integration.

Functional cohesion, synchrony and metastability were higher in provincial than connector RSNs. This dynamic profile suggests that provincial networks show both high-levels of coordination and greater capacity for altering their network states in order to adapt to multiple and diverse external demands. On the other hand, the DMN and CEN, which are implicated mostly in internal and goal-directed processing^5,20^, had the lowest metastability and synchrony. The salience network (SAL), which is considered important in tuning neural activity from internal to salient external stimuli^7^, occupied an intermediate position. The lower metastability of the internally-guided networks may reflect their role as a stable core of mental processing while their lower synchrony may reflect the ability of their component regions to show asynchronous activity profiles, potentially enabling more diverse functions.

We used the Kuramoto model of coupled oscillators^21^ to investigate the alignment of simulated and empirical dynamic RSN properties. There are alternative computational models that could be used to simulate the dynamics of both amplitudes and phases of oscillations, such as the Hopf model^22–24^, the Wilson-Cowan model^25,26^, the FitzHugh-Nagumo model^27^, and the Jansen-Rit model^28^. We chose the Kuramoto model because of its relative simplicity and ability to capture essential aspects of phase dynamics^12,16,29–33^. Additionally, the Kuramoto model has been successfully used in neuroscience research for the purpose of modeling slow^29,34,35^ and fast^12,16,33,36–39^ cortical oscillations. Here, we sought to evaluate the performance of the model with fast local gamma-band oscillations, in agreement with experimental^40–43^ and theoretical models of neural networks^44^. We found that the alignment between empirical and simulated metastability and synchrony occurred within anatomical coupling strengths (*k*) ranging from 5 to 12. Simulated and empirical synchrony were most closely aligned around the point of criticality, defined as the point of transition between asynchronous (dominated by noise that prevents information flow) and globally synchronized states (that are static and have no behavioral value). These findings confirm our previous report that empirical and simulated fMRI data align within the critical range of coupling strengths^33^, and are in line with the model of Kitzbichler and colleagues who proposed that simulated time series generate behaviors that most resemble those of empirical datasets when their global coupling strength reaches criticality^45^.

In summary, this study demonstrates that functional and dynamic RSN properties are connected and that the Kuramoto model provides a viable option for the investigation of the dynamic properties of empirical RSNs.

## Materials and Methods

### Participants

Thirty healthy adults (19–47 years; mean age = 27.2 years; 16 females) were recruited following advertisements in the local press. They were interviewed to exclude any past or current medical disorder or head trauma and any psychiatric disorder based on the Mini-International Neuropsychiatric Interview (M.I.N.I.)^46^. Their mean intelligence quotient (IQ) was 119.3 (SD = 14.9) based on the Wechsler Abbreviated Scale of Intelligence (WASI-II)^47^. The study protocol was approved by the Institutional Review Board of the Icahn School of Medicine at Mount Sinai (ISMMS). All experimental procedures were performed in accordance with standard protocols for magnetic resonance imaging studies. All participants provided written informed consent.

### Neuroimaging data acquisition

All imaging data were acquired on a Siemens Magnetom Skyra 3 T scanner (Erlangen, Germany) at the ISMMS. Participants were asked to keep their eyes open during the scan. Rs-fMRI data were acquired with a multi-band accelerated gradient-echo echo-planar imaging (EPI) sequence (TR = 1000 ms; TE = 35 ms; FOV = 228 × 228 mm; matrix = 95 × 95; 2.1 mm isotropic voxels; FA = 60°; 70 slices). T1-weighted structural MRI images were obtained with a 3-D magnetization-prepared rapid gradient-echo (MPRAGE) (TR = 2400 ms; TE = 2.07 ms; TI = 1000 ms; FOV = 256 × 256 mm; 0.8 mm isotropic voxels; FA = 8°; in-plane acceleration factor = 2). DTI data were acquired using a single-shot spin-echo EPI sequence with a multi-band factor of 3 and monopolar gradient pulse (TR = 3650 ms; TE = 85 ms; FOV = 208 × 176 mm; matrix = 116 × 98; 1.8 mm isotropic voxels; FA = 80°; 75 slices). The diffusion sensitizing gradients with a b-value of 1200 s/mm^2^ were applied in 64 non-collinear directions (left-to-right phase encoding) with 5 non-diffusion weighted (b_0_) images. This scan was repeated with phase encoding gradients of reverse polarity to correct for b_0_- and eddy current-induced distortions and to improve signal-to-noise ratio.

### Empirical fMRI data analyses

Preprocessing of the rs-fMRI data was performed using Statistical Parametric Mapping (SPM) software (SPM12; http://www.fil.ion.ucl.ac.uk/spm/) and the Data Processing Assistant for rs-fMRI toolbox^48^. Steps included slice timing correction, re-alignment, coregistration of functional images to the anatomical T1-weighted images followed by spatial normalization to the Montreal Neurological Institute (MNI) stereotaxic standard space, and spatial smoothing with a Gaussian kernel with a full-width half-maximum of 6 mm. We then applied a wavelet despiking technique for denoising signal transients related to small amplitude head movements (<1 mm)^49^. In all participants, head motion was less than 2 mm of displacement and 0.5 degrees of rotation in any direction. We regressed out 24 motion re-alignment parameters (6 motor parameters, 6 temporal derivatives, 6 quadratic terms, and 6 quadratic expansions of the derivatives of motion estimates) to compensate for head movement inside the MRI scanner^50^. Linear trends were removed and white matter and cerebrospinal fluid (CSF) signals were regressed out from the data using a component based noise reduction method (CompCor, 5 principal components)^51^. These preprocessing steps minimized the impact of head motion and physiological noise on the rs-fMRI data. Subsequently, for each subject we averaged the blood-oxygen-level dependent (BOLD) time series from each of 90 brain regions of interest (ROIs) (45 in each hemisphere) defined using the Automated Anatomical Labeling (AAL) template^52^ (Supplemental Table S1).

The BOLD time series from each of the 90 ROIs was filtered to extract frequency-band specific fMRI signals using the maximal overlap discrete wavelet transform^53,54^. Following the approach of Zhang *et al*.^53^, we utilized a wavelet filter of Daubechies Least Asymmetric with a wavelet length of 8 to extract the wavelet coefficients. We focused on scale 4 of the wavelet decomposition, corresponding to the frequency band of 0.03–0.06 Hz^55–57^. The Hilbert transform was applied to the wavelet-filtered fMRI signals to compute the associated analytical signals. The analytic signal represents a narrowband signal, $s(t)$, in the time domain as a rotating vector with an instantaneous phase, $\phi (t)$, and an instantaneous amplitude, $A(t)$, i.e., $s(t)=A(t)\cos (\phi (t))$. The phase and the amplitude are given by the argument and the modulus, respectively, of the complex signal $z(t)$, given by $z(t)=s(t)+i.H[s(t)]$, where $\,i$ is the imaginary unit and $H[s(t)]$ is the Hilbert transform of $s(t)$ ^34,57,58^. Subsequently, the first and last 10 time steps were discarded to avoid border effect inherent to the Hilbert transform, such that in the following T = 390 seconds^34^.

### Empirical DTI data analyses

We analyzed the DTI data using an in-house image processing pipeline that combines tools from the FSL’s diffusion toolbox (FDT; https://fsl.fmrib.ox.ac.uk/fsl/fslwiki/FDT)^59^, the DSI Studio^60^ and custom routines written in Matlab (Mathworks, Natick, MA, USA). Artifacts and spatial distortions due to eddy currents and head motion were corrected by deploying an affine registration between diffusion-weighted images and non-diffusion-weighted (b_0_) images. Non-brain tissue was removed using the brain extraction tool (BET) with a fractional intensity threshold of 0.1. Then, diffusion eigenvectors, eigenvalues, and fractional anisotropy (FA) for each voxel were computed using the *dtifit* function. Voxel-wise estimates of the angular distribution of local tract direction were calculated using the *bedpostx* function, which estimated a 2-fiber model using Markov Chain Monte Carlo sampling. Next, a total of 100,000 whole brain tracks were obtained using a deterministic fiber tracking algorithm^60^. The anisotropy threshold and step size were 0.1 and 0.9 mm, respectively, determined automatically in DSI Studio (http://dsi-studio.labsolver.org/). The angular threshold was 60°. Fiber tracks with lengths less than 10 mm were discarded to prevent the tracking process from being overwhelmed by short association fibers. The parcellation was performed by warping the subject space to a standard space using nonlinear registration^61^. For each subject, the AAL template was used to parcellate the entire brain into 90 ROIs identical to those used for the rs-fMRI data. We generated a symmetric weighted structural connectivity matrix representing the density of white matter fiber tracts (streamline density) connecting any two ROIs and a tract length matrix representing the average length across all the fibers connecting them. The individual structural connectome data of all participants were averaged to obtain a group-averaged structural connectivity matrix and a tract length matrix. The edge weight of the structural connectivity matrix, representing the structural connectivity network organization of the brain, was normalized by the volume of the interconnected ROIs to account for different size of the ROIs^62^.

### Computational modeling

We simulated phase time series using the Kuramoto model constrained by the empirical structural connectivity. Each of the 90 AAL-defined ROIs was considered as an oscillator. The phase at each ROI over time ${\theta }_{n}(t)$ is described by a set of coupled differential equation^16,21,38,63^:

$$\frac{d{\theta }_{n}(t)}{dt}={\omega }_{n}+k\sum _{p=1}^{N}{C}_{np}\,\sin ({\theta }_{p}(t-{D}_{np})-{\theta }_{n}(t))$$

where ${\theta }_{n}$ and ${\omega }_{n}$ denote the phase and intrinsic frequency of region $n$. *N* is the total number of regions. $k$ is the global coupling strength which scales all connections’ strength. ${C}_{np}$ is the relative coupling strength between region $n$ and region $p$ based on the empirical structural connectivity matrix. ${D}_{np}$ (ms) is the propagation delay matrix, determined using ${D}_{np}={L}_{np}/v,$ with ${L}_{np}$ (mm) the empirical tract length matrix and $v$ (m/s) the mean conduction velocity, such that $v=mean(\bar{L})/\tau$, i.e., the mean tract length divided by the mean delay.

We ran the Kuramoto simulations with a mean delay of $\tau$ = 6 ms, corresponding to a mean conduction velocity of 9.6 m/s (mean tract length = 57.3 mm), which is the midpoint within the range of physiologically realistic values estimated at 5–20 m/s^64^. Phases were initialized randomly. We set the intrinsic frequencies to be uniformly distributed with mean = 60 Hz and SD = 1 Hz, corresponding to oscillations within the gamma frequency range^16,38,65^, as gamma local field potential (LFP) power is coupled to the BOLD fMRI signal and is considered representative of the overall neuronal activity^40–43^. Simulations were run for 410 seconds (first 20 seconds were discarded to remove transient effects, such that T = 390 seconds identical to time lengths of empirical fMRI data) with a time-step of 0.1 ms for a range of global coupling strengths (1 ≤ *k* ≤ 25 at a resolution of 1) using an Euler scheme.

### Functional and dynamic network properties

In each subject, ROIs were assigned to the DMN, CEN, SAL, SMN, VN, and AN based on functional templates available through the Functional Imaging in Neuropsychiatric Disorders Lab at Stanford University, USA (https://findlab.stanford.edu/functional_ROIs.html) (Supplemental Table S2). Within-network functional connectivity for each RSN was computed by averaging the Pearson’s correlation between the time series of all the voxels of the ROIs assigned to each particular network. For between-network functional connectivity, we first calculated an average time series within each RSN (as described above) and then computed the Pearson’s correlation between the time series of each network and all the other networks (see also Supplementary Information (SI), section 2.3). These computations resulted in 6 within-network and 6 between-network functional connectivity measures per participant that were then Fisher Z-transformed. Network cohesion (i.e., within-network connectivity) was defined as the mean strength of the functional connectivity between the regions of a given network and reflects functional cohesion; while network integration (i.e., between-network connectivity) was defined as the mean strength of the functional connectivity between one network and all the other networks, reflecting functional integration^9^.

To evaluate the dynamic properties of each RSN, we computed the order parameter $R(t)$, defined as

$$R(t)=|\,\frac{1}{N}\sum _{n=1}^{N}{e}^{i{\phi }_{n}(t)}\,|$$

where *N* is the total number of regions within each RSN and ${\phi }_{n}(t)$ is the instantaneous phase of regional mean BOLD time series at region *n*. The temporal fluctuation of the order parameter $R(t)$ for each participant is shown in Supplementary Figure S1. We considered the mean of the order parameter $R(t)$ across time, as an index of synchrony and the standard deviation of the $\,R(t)$, as an index of metastability^12,16,17,33^. With regards to the simulated data, we note that first-order Kuramoto models do not exhibit true metastability^36^ but we follow the convention of using the term metastability to refer to the standard deviation of $R(t)$ ^16,17,19^. We compared metastability and synchrony resulting from the Kuramoto model against the empirical data. The model error was quantified in terms of the absolute difference between the simulated and empirical values for a range of coupling strengths.

### Reliability analyses

We undertook a number of reliability analyses as detailed in Supplementary Information (SI) to examine the effect of (a) rs-fMRI scan duration (volumes/time points) on metastability and synchrony (SI section 2.1); (b) setting the intrinsic frequencies of the oscillators to be normally distributed with mean 0.045 Hz and SD = 0.01 Hz, corresponding to the center frequency of empirical fMRI signal (SI section 2.2); (c) using phase-locking value-based connectivity as an alternate method for defining RSN cohesion and integration conditions (SI section 2.3); (d) estimating the metastability and synchrony using rs-fMRI datasets from a sample of 30 participants of the Human Connectome Project (SI section 2.4) that were demographically matched to the original study sample. Finally, to ensure robustness of the simulation results, we conducted 5 additional runs of the Kuramoto simulation with varying random initial conditions (SI section 2.5).

### Data availability

The datasets generated during the current study are available from the corresponding author on reasonable request.

## Electronic supplementary material

Supplemental Information
