# Supplementary material for: Linking functional connectivity and dynamic properties of resting-state networks
Source: Sci Rep. 2017 Nov 30;7:16610. doi: 10.1038/s41598-017-16789-1 (PMC5709368; doi:10.1038/s41598-017-16789-1)
Supplement: Supplementary file 1 — Supplemental Information [file 41598_2017_16789_MOESM1_ESM.doc]

**Supplemental Information**

**for manuscript**

**Linking functional connectivity and dynamic properties of resting-state networks**

Won Hee Lee, Sophia Frangou*

Department of Psychiatry, Icahn School of Medicine at Mount Sinai, New York, NY 10029, USA

* Corresponding Author

Department of Psychiatry, Icahn School of Medicine at Mount Sinai, 1425 Madison Avenue

New York, NY 10029, USA; E-mail: [sophia.frangou@mssm.edu](mailto:sophia.frangou@mssm.edu); Tel: +1-212-659-1668

**1. Temporal dynamics of coherence in resting-state networks**

Figure S1 illustrates temporal fluctuations of the order parameter amplitude *R*(*t*) for each individual for each of the six resting-state networks. Color-coded thick lines represent the mean order parameter amplitudes averaged across all participants over time.

**
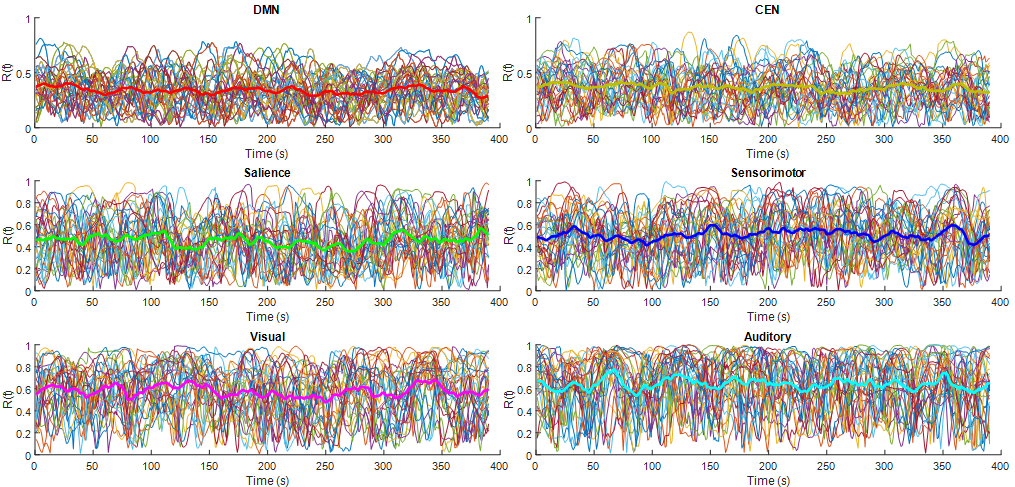
**

**Figure S1**. Temporal fluctuations of the order parameter amplitude *R*(*t*) for each resting-state networks (RSN) for each individual. Color-coded thick lines indicate the mean order parameter amplitudes averaged across all participants over time. DMN = default mode network, CEN = central executive network.

**2. Reliability analyses**

**2.1 Reliability of results as a function of the number of the rs-fMRI time points/volumes**

To test reliability, we estimated phase-locking values (PLV) from the first (9 periods of oscillations) and the second half (9 periods of oscillations) of the resting-state fMRI (rs-fMRI) dataset. The original scan duration presented in the main manuscript had 410 time points at a TR = 1000 ms, corresponding to a total duration of 6 min 50 s (18 periods of oscillations). Group-average PLVs estimated from each half of all the scans (i.e., short scan duration) were compared to the original group-average PLV (Figure S2). The results were closely matched in terms of mean squared error (MSE), corresponding to 0.0048 and 0.0046 for the first half and the second half of the data, respectively. These results suggest that PLV from relatively short scan duration is robust and reliable when estimating PLV. Next, using these datasets, we computed empirical metastability and synchrony for each RSN (Figure S3). Analysis of variance, followed by a post-hoc analysis, revealed that metastability (all F_2_ > 0.31; *p* > 0.282) and synchrony (all F_2_ > 0.29; *p* > 0.440) are not statistically different between the rs-fMRI dataset with different number of the rs-fMRI time points (i.e., scan duration) for all RSNs.


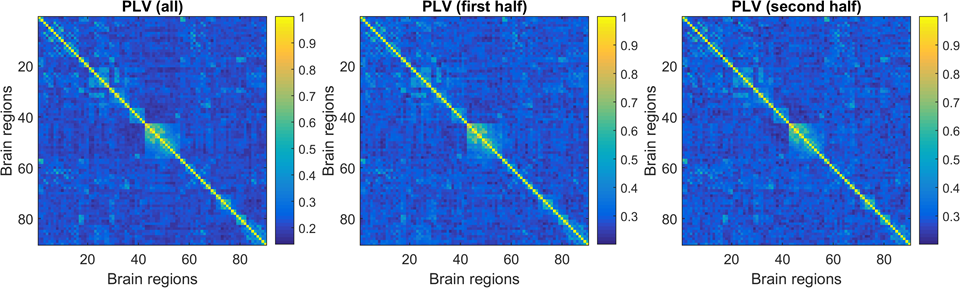


**Figure S2**. Phase-locking value (PLV) averaged across subjects estimated from the original scan (all), the first half and the second half of the resting-state fMRI dataset.


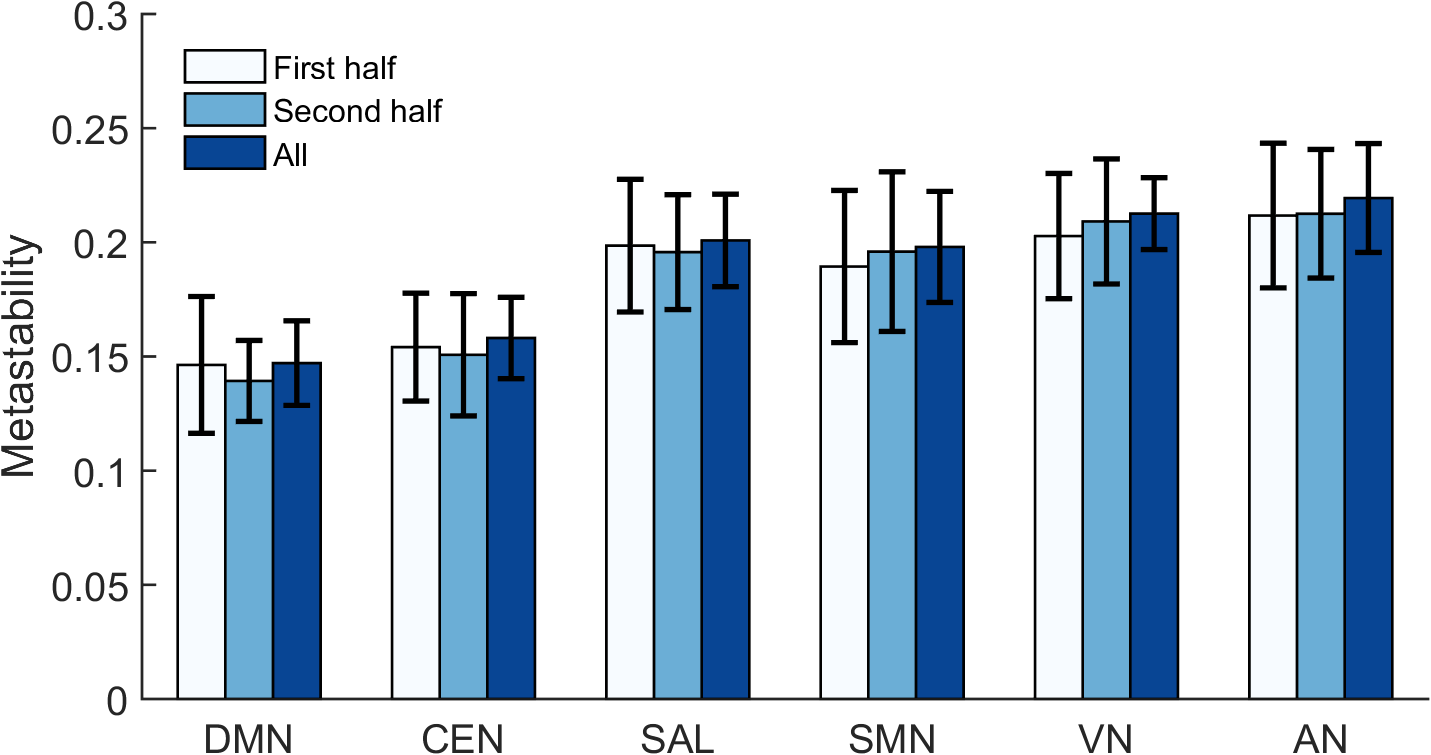


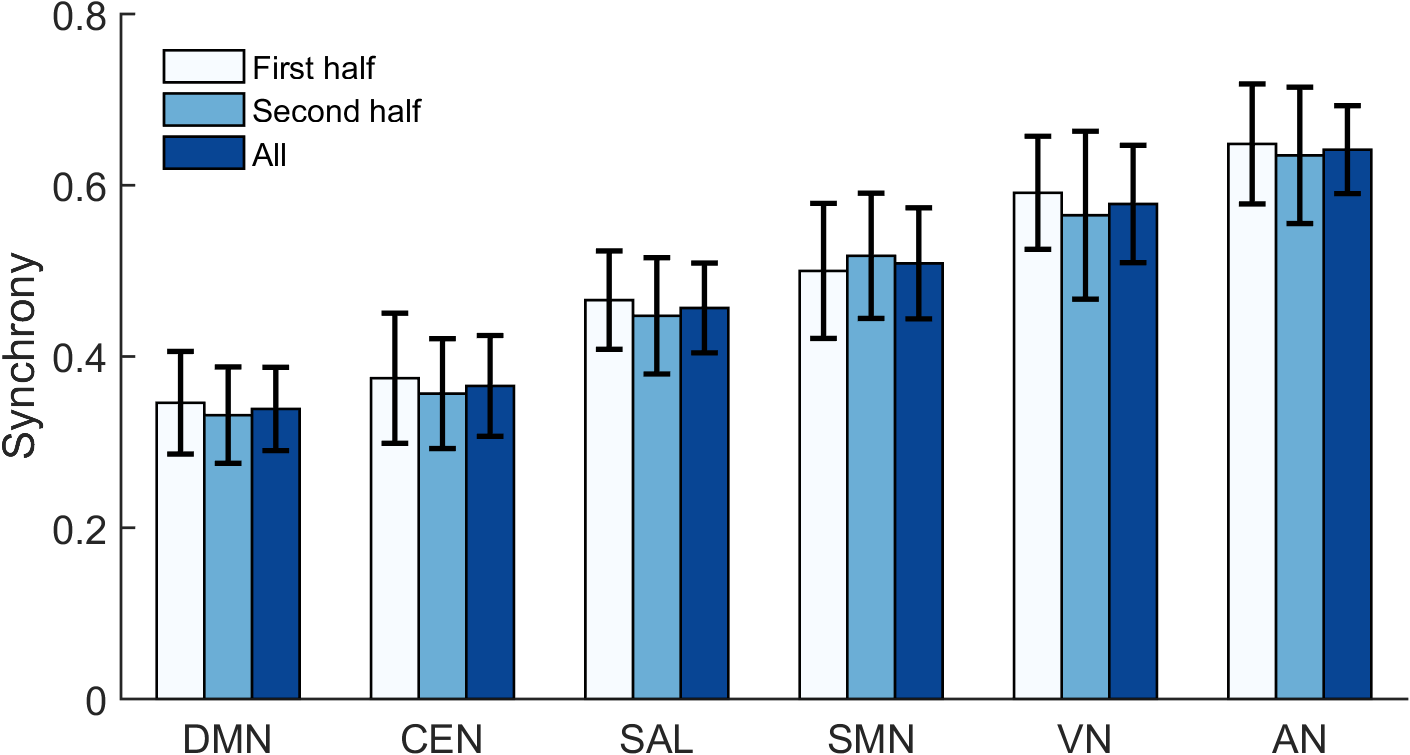


**Figure S3**. Empirical metastability and synchrony estimated from the original (all), the first half and the second half of the resting-state fMRI dataset for each RSN. Bars and error bars indicate the mean and standard deviation across subjects, respectively. DMN = default mode network, CEN = central executive network, SAL = salience network, SMN = sensorimotor network, VN = visual network, AN = auditory network.

**2.2 Reliability of results given a “slow” Kuramoto model**

We performed the Kuramoto simulation with slow oscillators^1^ setting their intrinsic frequencies to be normally distributed with mean 0.045 Hz and SD = 0.01 Hz, corresponding to the center frequency of empirical fMRI signal. The slow Kuramoto simulation yielded comparable metastability and synchrony results as a function of coupling strength (Figure S4). Based on this, we have retained our original analyses in the main manuscript.


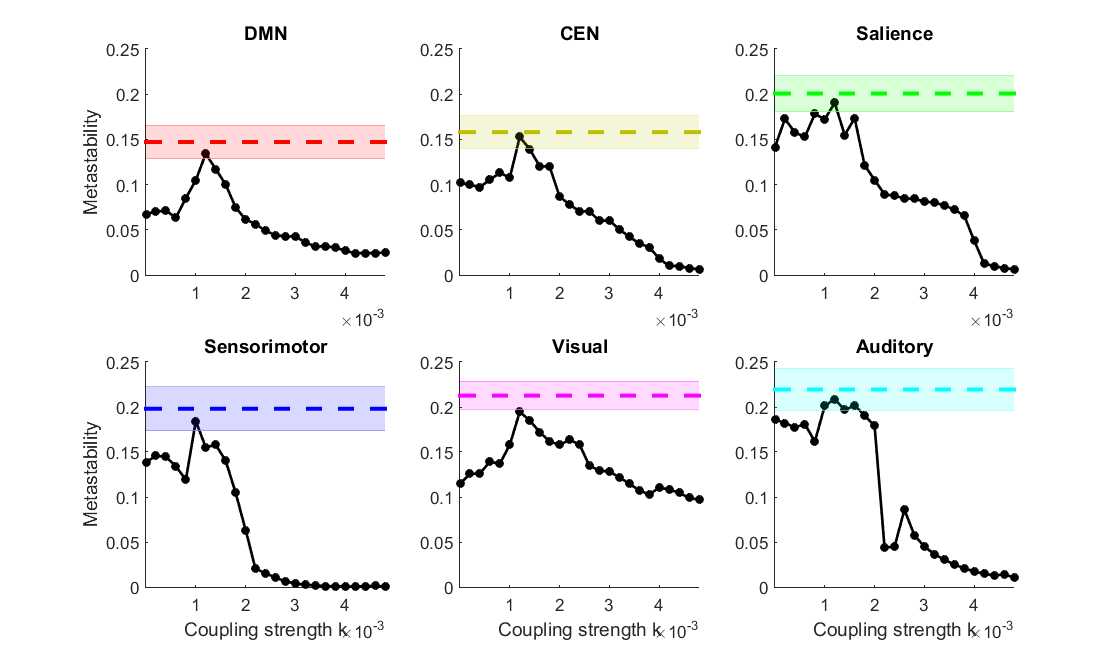


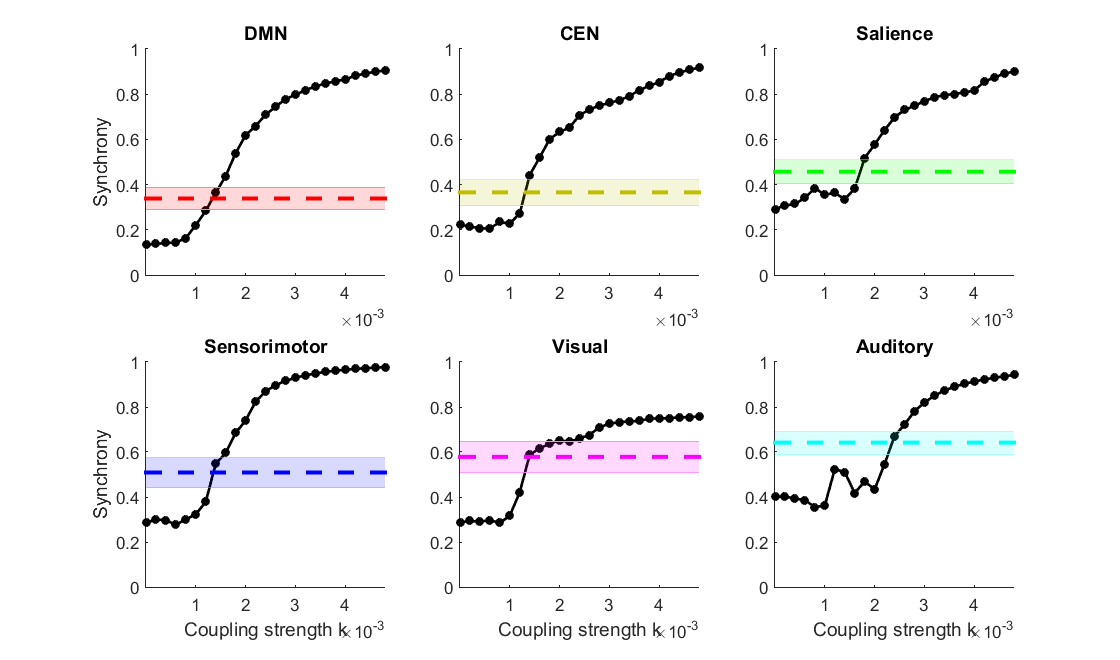


**Figure S4**. Simulated metastability (top) and synchrony (bottom), resulting from s*low* Kuramoto model, on the structural coupling strength *k* and comparison with empirical metastability and synchrony values estimated using phase-transformed fMRI time series of 30 healthy subjects for each of the six resting-state networks (RSNs). DMN = default mode network, CEN = central executive network.

**2.3 Comparison of correlation- and PVL-based functional connectivity measures**

In each subject, AAL-derived regions-of-interest were assigned to the DMN, CEN, SAL, SMN, VN, and AN based on functional templates available through the Functional Imaging in Neuropsychiatric Disorders Lab at Stanford University, USA (https://findlab.stanford.edu/functional_ROIs.html) (Supplementary Table S2). Within-network functional connectivity for each RSN was computed by averaging the Pearson’s correlation between the time series of all the voxels of the ROIs assigned to each particular network. For between-network functional connectivity, we first calculated an average time series within each RSN (as described above) and then computed the Pearson’s correlation between the time series of each network and all other networks. These computations resulted in 6 within-network and 6 between-network functional connectivity measures per participant that were then Fisher Z-transformed.

We also computed connectivity $F_{np}$ between any pair of regions *n* and *p* using the phase-locking value (PLV) that is the average of the instantaneous phase relations over time, given as^2^:

$$F_{np}=\left|\frac{1}{T}\sum_{t=1}^{T} e^{i[\phi_{n}(t)-\phi_{p}(t)]}\right|$$

The phase-locking value ranges from 0 (complete phase independence) to 1 (perfect phase-locking).

The cohesion coefficient of region *n* with respect to system S (i.e., RSN), $W_{n}^{S}$, is defined as:

$$W_{n}^{S}=\frac{1}{n_{S}}\sum_{p\in S} F_{np}$$

where $n_{S}$ is the number of regions in a given network S. Network integration (i.e., between-network connectivity) was defined as the mean strength of functional connectivity between the ROIs of one network and those of all the other networks^3^. The integration coefficient of region *n* with respect to system S, $I_{n}^{S}$, is defined as:

$$I_{n}^{S}=\frac{1}{N-n_{S}}\sum_{p\notin S} F_{np}$$

where *N* is the total number of all brain ROIs and $n_{S}$ is the number of regions in a given network S.

We compared the correlation-based and PLV-based matrix (averaged across subjects; see Figures S5A and S5B) by calculating the correlation coefficient between corresponding elements of the upper triangular part of the two matrices. Figure S5C shows that the relationship between the correlation-based and PLV-based functional connectivity is not linear. Figure S6 shows the relationship between the dynamic properties of the empirical RSNs and the functional connectivity, either based on the Pearson correlation (Figure S6A) or phase-locking value (Figure S6B), indicating that the results are comparable.


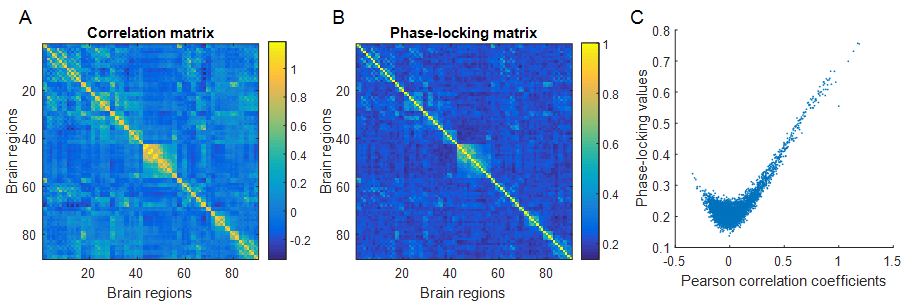


**Figure S5**. Group-average (A) Pearson correlation matrix, (B) phase-lock value matrix and (C) comparison between the two functional connectivity matrices.


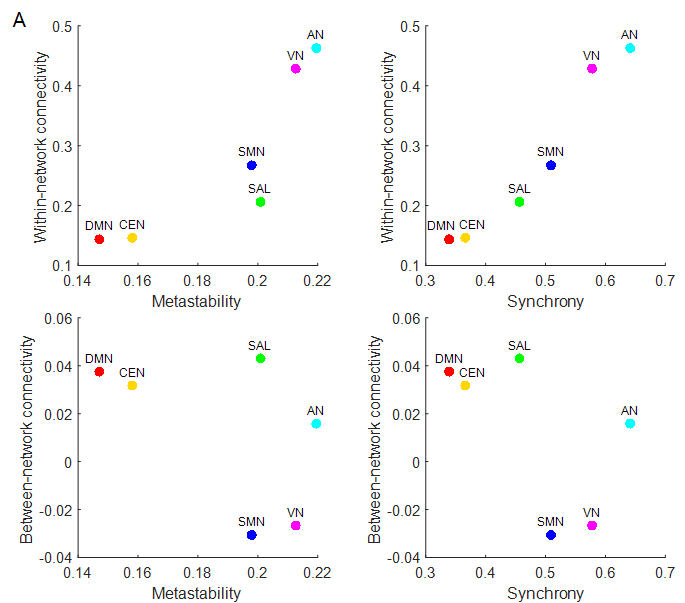


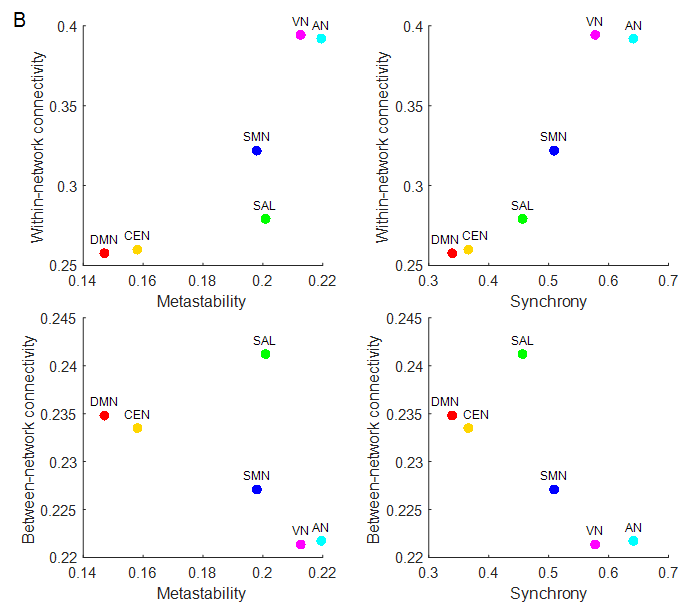


**Figure S6**. Cartographic representation of the functional connectivity and dynamic properties of the empirical resting-state networks: (A) correlation-based functional connectivity and (B) phase-locking value-based connectivity. Each network is represented in a position defined by its average values for these measures. DMN = default mode network, CEN = central executive network, SAL = salience network, SMN = sensorimotor network, VN = visual network, AN = auditory network.

**2.4 Influence of scan duration on metastability and synchrony using the Human Connectome Project (HCP) dataset**

We examined the influence of scan duration on metastability and synchrony using rs-fMRI data from the Human Connectome Project (HCP; https://www.humanconnectome.org). We selected data from the first 30 HCP participants that matched the original study sample in age (mean age = 27.2 years) and sex (16 females). The HCP rs-fMRI scan has a total duration of 14 min 24 s at a TR = 720 ms, corresponding to 1200 time points and 40 periods of oscillations. Detailed parameters of the HCP rs-fMRI acquisition sequence are outlined in Smith et al. (2013)^4^. To fully capture the effect of scan duration, we computed the metastability and synchrony of each RSN for the entire duration of the scan (14 min 24 s; 40 periods of oscillations) and at 570 time points (6 min 50 s; 18 periods of oscillations) and at 855 time points (10 min 16 s; 28 periods of oscillations) (Figure S7). The subdivisions were extracted from the beginning of the fMRI time series to each of the selected time points. The time series extracted from the HCP data set at 570 time points corresponds to a total duration of our original study dataset (410 time points at a TR = 1000 ms; 18 periods of oscillations). Two separate analyses of variance with metastability (all F_2_ < 0.29; *p* > 0.75) or synchrony (all F_2_ < 0.23; *p* > 0.79) as dependent variables and time points (570, 855, and 1200 time points) as independent factors did not identify significant differences (Supplementary Table S4). Bonferroni corrected post-hoc pairwise comparisons also showed that metastability and synchrony were comparable between the rs-fMRI dataset with different number of time points for all RSNs (Supplementary Table S5).


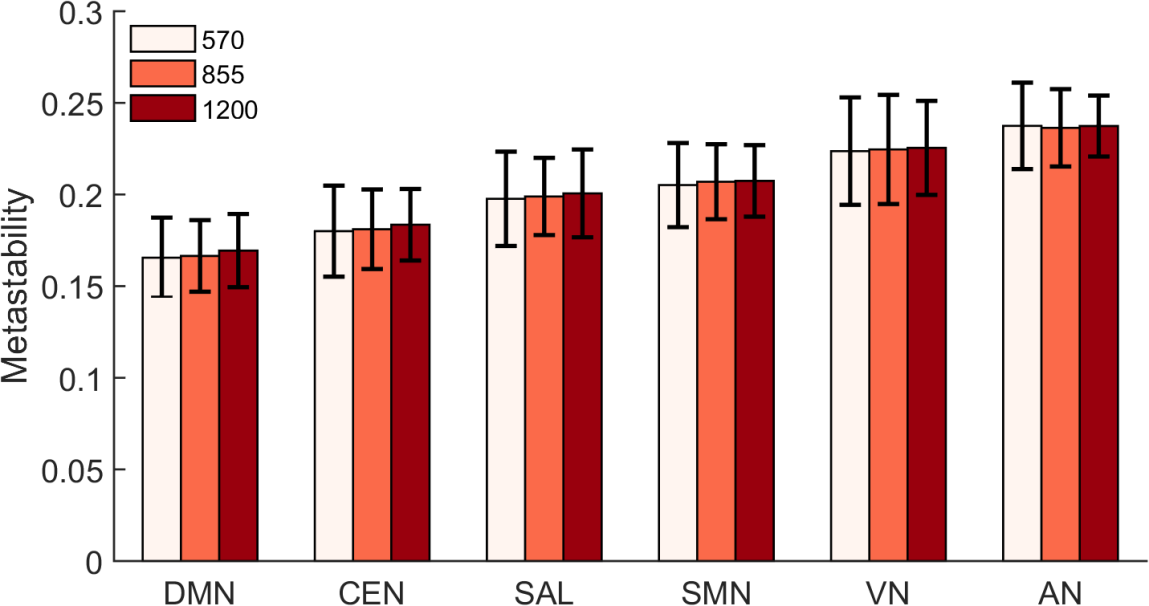


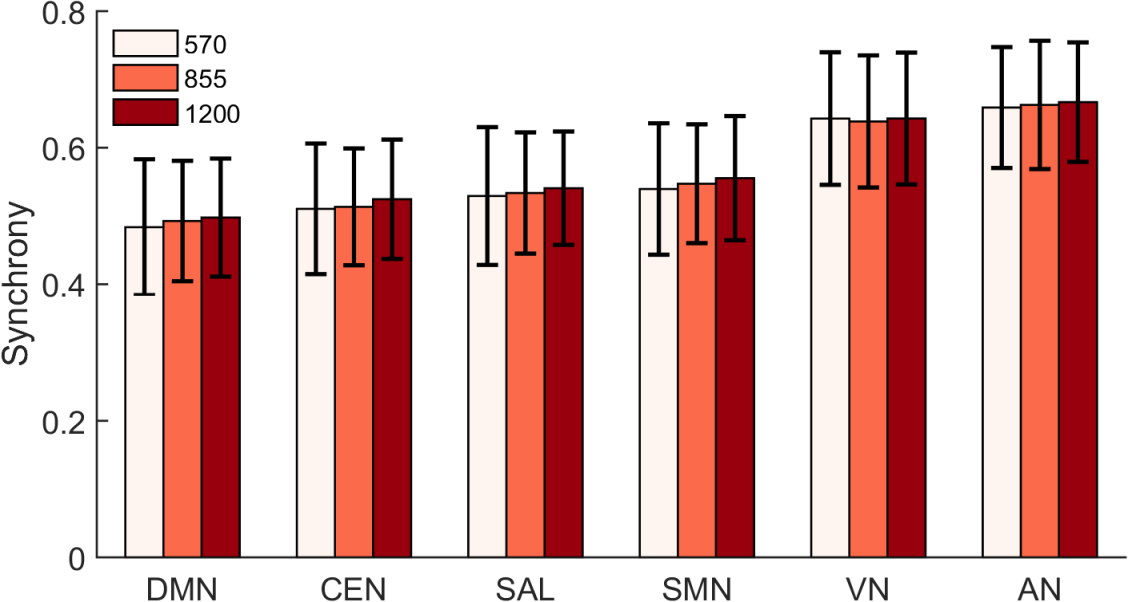


**Figure S7**. Empirical metastability and synchrony for each RSN estimated from the rs-fMRI data of the Human Connectome Project at different number of time points/volumes (570, 855, and 1200 time points). Bars and error bars correspond respectively to the mean and standard deviation across all participants. DMN = default mode network, CEN = central executive network, SAL = salience network, SMN = sensorimotor network, VN = visual network, AN = auditory network.

**2.5 Dependence of results on random initial conditions in the Kuramoto model**

To ensure robustness of the simulation results on random initial conditions, we conducted additional 5 runs of the Kuramoto simulations with varying initial conditions. Figure S8 shows the behavior of the Kuramoto model averaged across 5 runs for both metastability (top, red) and synchrony (bottom, green) as a function of coupling strength *k* for each of the six resting-state networks (RSNs). We obtained similar behavior of the Kuramoto model with varying initial conditions for a range of coupling strengths. These results demonstrate minimal dependence of the results on the random initial conditions in the Kuramoto simulation and are reproducible.

**
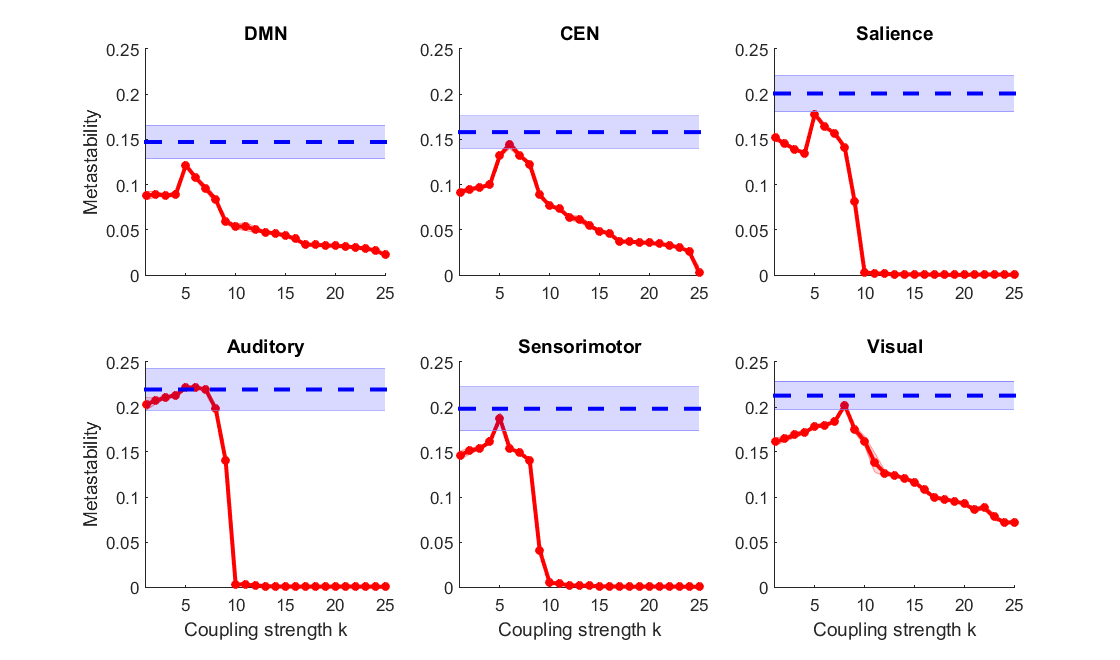
**

**
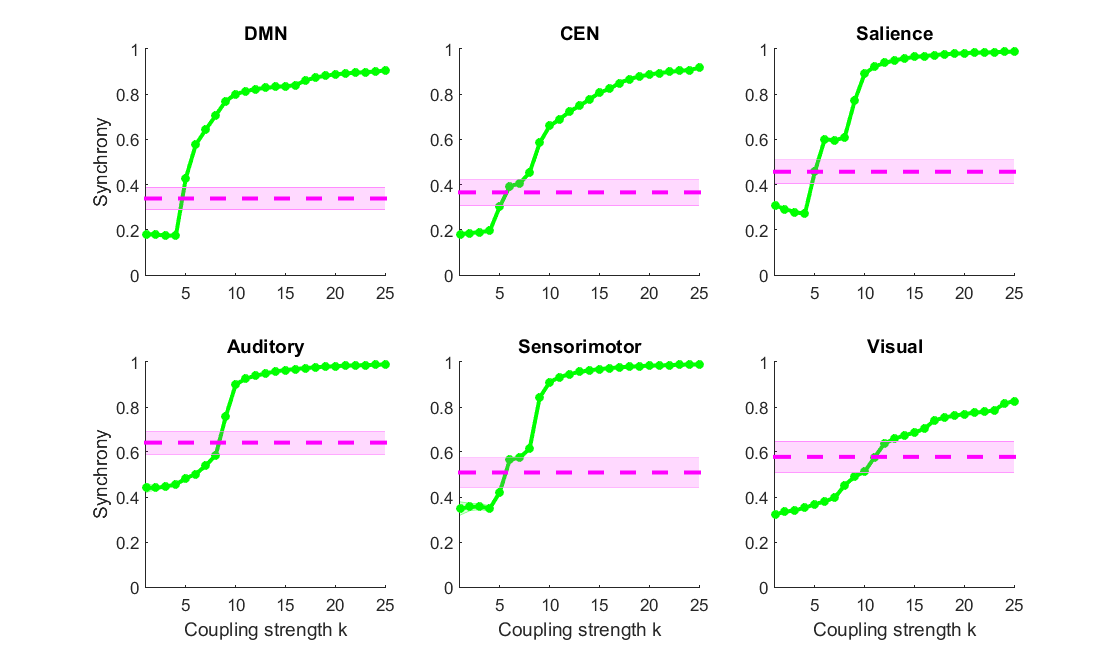
**

**Figure S8**. Dependency of simulated metastability (top, red) and synchrony (bottom, green), averaged across 5 runs of the Kuramoto simulations, on the structural coupling strength *k* and comparison with empirical metastability (top, blue) and synchrony (bottom, magenta) values estimated using phase-transformed fMRI time series of 30 healthy subjects for each of the six resting-state networks (RSNs). DMN = default mode network, CEN = central executive network.

**3**. Supplemental Tables

| **Table S1. Brain regions and abbreviations considered in resting-state networks from the Automated Anatomical Labeling (AAL) template (Tzourio-Mazoyer et al., 2002). The numerical indices indicate the order of the numeric identifiers of these brain regions in the left and right hemispheres.** | | | |
| --- | --- | --- | --- |
| **Region** | **Abbreviation** | **Left** | **Right** |
| Precental gyrus | PreCG | 1 | 2 |
| Superior frontal gyrus, dorsolateral | SFGdor | 3 | 4 |
| Superior frontal gyrus, orbital part | ORBsup | 5 | 6 |
| Middle frontal gyrus | MFG | 7 | 8 |
| Middle frontal gyrus, orbital part | ORBmid | 9 | 10 |
| Inferior frontal gyrus, opercular part | IFGoperc | 11 | 12 |
| Inferior frontal gyrus, triangular part | IFGtriang | 13 | 14 |
| Inferior frontal gyrus, orbital part | ORBinf | 15 | 16 |
| Rolandic operculum | ROL | 17 | 18 |
| Supplementary motor area | SMA | 19 | 20 |
| Olfactory cortex | OLF | 21 | 22 |
| Superior frontal gyrus, medial | SFGmed | 23 | 24 |
| Superior frontal gyrus, medial orbital | ORBsupmed | 25 | 26 |
| Gyrus rectus | REC | 27 | 28 |
| Insula | INS | 29 | 30 |
| Anterior cingulate and paracingulate gyri | ACG | 31 | 32 |
| Median cingulate and paracingulate gyri | DCG | 33 | 34 |
| Posterior cingulate gyrus | PCG | 35 | 36 |
| Hippocampus | HIP | 37 | 38 |
| Parahippocampal gyrus | PHG | 39 | 40 |
| Amygdala | AMYG | 41 | 42 |
| Calcarine fissure and surrounding cortex | CAL | 43 | 44 |
| Cuneus | CUN | 45 | 46 |
| Lingual gyrus | LING | 47 | 48 |
| Superior occipital gyrus | SOG | 49 | 50 |
| Middle occipital gyrus | MOG | 51 | 52 |
| Inferior occipital gyrus | IOG | 53 | 54 |
| Fusiform gyrus | FFG | 55 | 56 |
| Postcentral gyrus | PoCG | 57 | 58 |
| Superior parietal gyrus | SPG | 59 | 60 |
| Inferior parietal, but supramarginal and angular gyri | IPL | 61 | 62 |
| Supramarginal gyrus | SMG | 63 | 64 |
| Angular gyrus | ANG | 65 | 66 |
| Precuneus | PCUN | 67 | 68 |
| Paracentral lobule | PCL | 69 | 70 |
| Caudate nucleus | CAU | 71 | 72 |
| Lenticular nucleus, putamen | PUT | 73 | 74 |
| Lenticular nucleus, pallidum | PAL | 75 | 76 |
| Thalamus | THA | 77 | 78 |
| Heschl gyrus | HES | 79 | 80 |
| Superior temporal gyrus | STG | 81 | 82 |
| Temporal pole: superior temporal gyrus | TPOsup | 83 | 84 |
| Middle temporal gyrus | MTG | 85 | 86 |
| Temporal pole: middle temporal gyrus | TPOmid | 87 | 88 |
| Inferior temporal gyrus | ITG | 89 | 90 |

| **Table S2. Anatomical location of functional regions of interest (ROIs) to construct six resting-state networks (RSNs)** | |
| --- | --- |
| **RSN** | **Regions** |
| Default model network | Medial prefrontal cortex  Orbitofrontal cortex  Angular gyrus  Superior frontal gyrus (R)  Precuneus  Anterior cingulate cortex  Midcingulate cortex  Posterior cingulate cortex  Retrosplenial cortex  Middle frontal gyrus  Parahippocampal gyrus  Middle occipital gyrus  Thalamus  Hippocampus |
| Central executive network | Middle frontal gyrus  Superior frontal gyrus  Inferior parietal gyrus  Angular gyrus  Inferior frontal gyrus (L)  Orbitofrontal gyrus (L)  Superior parietal gyrus (L)  Precuneus (L)  Inferior temporal gyrus (L)  Middle temporal gyrus (L)  Thalamus (L)  Supramarginal gyrus (R)  Caudate (R) |
| Salience network | Middle frontal gyrus  Insula  Anterior cingulate cortex  Medial prefrontal cortex  Supplementary motor area |
| Auditory network | Superior temporal gyrus  Heschl’s gyrus  Thalamus (R) |
| Sensorimotor network | Precentral gyrus  Postcentral gyrus  Supplementary motor area  Thalamus |
| Visual network | Calcarine sulcus  Middle occipital gyrus  Superior occipital gyrus  Thalamus (L) |

| **Table S3. Multiple linear regression analyses between empirical metastability or synchrony and age, sex and IQ for each resting-state network** | | | | | | | | | | | |
| --- | --- | --- | --- | --- | --- | --- | --- | --- | --- | --- | --- |
| **RSN** | **Overall Model** | | **Age** | | | **Sex** | | | **IQ** | | |
|  | Adjusted R^2^ | p-value | B | p-value | 95% CI | B | p-value | 95% CI | B | p-value | 95% CI |
| **Metastability** | | | | | | | | | | | |
| **DMN** | 0.01 | 0.35 | 0.001 | 0.06 | 0,  0.003 | 0.012 | 0.16 | -0.008, 0.03 | 0 | 0.27 | 0, 0.001 |
| **CEN** | 0.02 | 0.30 | 0.001 | 0.35 | -0.001, 0.003 | -0.008 | 0.36 | -0.02,  0.006 | 1.077E-5 | 0.99 | 0,0 |
| **SAL** | 0.06 | 0.20 | -0.001 | 0.40 | -0.002,  0.001 | -0.009 | 0.30 | -0.02,  0.006 | -0.001 | 0.06 | -0.001,  1.026E-5 |
| **SMN** | -0.05 | 0.67 | 0 | 0.72 | -0.001,  0.002 | -0.02 | 0.80 | -0.02,  0.02 | 0 | 0.38 | -0.001,  0 |
| **VN** | -0.5 | 0.67 | 8.563E-5 | 0.84 | -0.001,  0.001 | -0.002 | 0.82 | -0.01,  0.01 | 0 | 0.27 | -0.001,  0 |
| **AN** | -0.007 | 0.43 | 0.001 | 0.23 | -0.001,  0.003 | 0.007 | 0.51 | -0.01,  0.03 | 0 | 0.16 | 0,  0.001 |
| **Synchrony** | | | | | | | | | | | |
| **DMN** | 0.002 | 0.40 | 0.001 | 0.35 | -0.002,  0.005 | 0.03 | 0.10 | -0.007,  0.08 | 0 | 0.51 | -0.001,  0.002 |
| **CEN** | -0.54 | 0.67 | 0.001 | 0.44 | -0.003,  0.005 | -0.01 | 0.65 | -0.06,  0.04 | 3.573E-5 | 0.95 | -0.002,  0.002 |
| **SAL** | 0.06 | 0.22 | -0.003 | 0.06 | -0.006,  0.001 | -0.03 | 0.15 | -0.08,  0.01 | -0.001 | 0.06 | -0.003,  0 |
| **SMN** | -0.01 | 0.45 | 0.002 | 0.42 | -0.002,  0.005 | 0.02 | 0.43 | -0.03,  0.08 | -0.001 | 0.26 | -0.002, 0.001 |
| **VN** | -0.07 | 0.80 | 0 | 0.90 | -0.004,  0.004 | -0.02 | 0.40 | -0.08,  0.03 | 0 | 0.85 | -0.002,  0.002 |
| **AN** | -0.04 | 0.63 | 0.001 | 0.50 | -0.003,  0.006 | -0.004 | 0.87 | -0.06,  0.05 | 0 | 0.57 | -0.002, 0.001 |
| **Within-Network Functional Connectivity** | | | | | | | | | | | |
| **DMN** | 0.02 | 0.33 | 0.002 | 0.12 | -0.001,  0.004 | 0.04 | 0.09 | -0.004,  0.08 | 0 | 0.54 | -0.001,  0.002 |
| **CEN** | 0.08 | 0.50 | 0.002 | 0.40 | -0.003,  0.007 | -0.020 | 0.53 | -0.08,  0.04 | 0 | 0.78 | -0.001,  0.002 |
| **SAL** | 0.10 | 0.12 | -0.005 | 0.06 | -0.01,  0 | -0.07 | 0.08 | -0.14,  0.006 | -0.002 | 0.06 | -0.004,  6.207E-5 |
| **SMN** | 0.09 | 0.47 | 0.002 | 0.44 | -0.003,  0.006 | 0.03 | 0.50 | -0.06,  0.11 | -0.001 | 0.23 | -0.003, 0.001 |
| **VN** | -0.06 | 0.72 | -0.001 | 0.75 | -0.008,  0.007 | -0.05 | 0.29 | -0.16,  0.04 | -0.001 | 0.71 | -0.004,  0.003 |
| **AN** | 0.02 | 0.31 | 0.001 | 0.82 | -0.009,  0.013 | -0.06 | 0.24 | -0.16,  0.05 | -0.001 | 0.43 | -0.004,  0.003 |
| **Between-Network Functional Connectivity** | | | | | | | | | | | |
| **DMN** | 0.07 | 0.75 | 0.001 | 0.50 | -0.002,  0.003 | -0.001 | 0.93 | -0.02,  0.02 | -4.055E-5 | 0.93 | -0.001,  0.001 |
| **CEN** | -0.11 | 0.95 | 0 | 0.81 | -0.002,  0.002 | 0 | 0.99 | -0.03,  0.02 | -2.760E-6 | 0.99 | -0.001, 0.001 |
| **SAL** | -0.06 | 0.74 | 0.001 | 0.44 | -.0002,  0.003 | -0.005 | 0.68 | -0.037,  0.02 | 4.848E-5 | 0.89 | -0.001,  0.001 |
| **SMN** | -0.07 | 0.79 | 0.001 | 0.65 | -0.002,  0.004 | 0.01 | 0.39 | -0.02,  0.05 | 0 | 0.52 | -0.001,  0.002 |
| **VN** | -0.04 | 0.62 | 0.001 | 0.44 | -0.003,  0.006 | 0.02 | 0.32 | -0.03,  0.07 | 0 | 0.66 | -0.001,  0.001 |
| **AN** | 0.09 | 0.13 | 0.001 | 0.46 | -0.002,  0.003 | -0.005 | 0.73 | -0.04,  0.03 | -0.001 | 0.22 | -0.002,  0 |
| B=unadjusted regression coefficient; CI= confidence interval; CI are shown as (lower, upper bound); IQ=Intelligence Quotient; DMN = default mode network; CEN = central executive network; SAL = salience network; SMN = sensorimotor network; VN = visual network; AN = auditory network; all analyses were conducted using bootstrapping (n=1000); separate models were computed metastability, synchrony , within- and between-network functional connectivity for each network; functional connectivity was computed using standard methods based on Pearson’s correlation | | | | | | | | | | | |

| **Table S4. Analysis of variance of metastability and synchrony for each resting-state network (RSN) estimated from the rs-fMRI data at 570 time points (6 min 50 s), 855 time points (10 min 16 s), and 1200 time points (14 min 24 s)** | | | | |
| --- | --- | --- | --- | --- |
| **RSN** | **SS** | **MS** | **F** | **p-value** |
| **Metastability** | | | | |
| **DMN** | 0.00024 | 0.00012 | 0.29 | 0.751 |
| **CEN** | 0.00020 | 9.77E-05 | 0.20 | 0.819 |
| **SAL** | 0.00013 | 6.55E-05 | 0.12 | 0.889 |
| **SMN** | 8.79E-05 | 4.39E-05 | 0.10 | 0.905 |
| **VN** | 4.69E-05 | 2.35E-05 | 0.03 | 0.971 |
| **AN** | 2.18E-05 | 1.09E-05 | 0.03 | 0.975 |
| **Synchrony** | | | | |
| **DMN** | 0.00304 | 0.00152 | 0.18 | 0.834 |
| **CEN** | 0.00331 | 0.00165 | 0.21 | 0.814 |
| **SAL** | 0.00199 | 0.00099 | 0.12 | 0.887 |
| **SMN** | 0.00378 | 0.00189 | 0.23 | 0.798 |
| **VN** | 0.00037 | 0.00019 | 0.02 | 0.980 |
| **AN** | 0.00094 | 0.00047 | 0.06 | 0.944 |
| SS = sum of squares; MS = mean square; DMN = default mode network; CEN = central executive network; SAL = salience network; SMN = sensorimotor network; VN = visual network; AN = auditory network. | | | | |

| **Table S5. Bonferroni corrected post-hoc pairwise comparisons in metastability and synchrony of each resting-state network (RSN) estimated from the rs-fMRI data at 570 time points (6 min 50 s), 855 time points (10 min 16 s), and 1200 time points (14 min 24 s)** | | | | | | |
| --- | --- | --- | --- | --- | --- | --- |
| **RSN** | **Group (I)** | **Group (J)** | **MD (I-J)** | **95% CI (lower, upper bound)** | | **p-value** |
| **Metastability** | | | | | | |
| DMN | 570 | 855 | -0.001 | -0.013, 0.012 | 1 | |
|  | 570 | 1200 | -0.004 | -0.016, 0.009 | 1 | |
|  | 855 | 1200 | -0.003 | -0.016, 0.010 | 1 | |
| CEN | 570 | 855 | -0.001 | -0.015, 0.013 | 1 | |
|  | 570 | 1200 | -0.003 | -0.017, 0.010 | 1 | |
|  | 855 | 1200 | -0.002 | -0.016, 0.011 | 1 | |
| SAL | 570 | 855 | -0.001 | -0.016, 0.014 | 1 | |
|  | 570 | 1200 | -0.003 | -0.018, 0.012 | 1 | |
|  | 855 | 1200 | -0.002 | -0.017, 0.013 | 1 | |
| SMN | 570 | 855 | -0.002 | -0.015, 0.011 | 1 | |
|  | 570 | 1200 | -0.002 | -0.015, 0.011 | 1 | |
|  | 855 | 1200 | -0.0004 | -0.014, 0.013 | 1 | |
| VN | 570 | 855 | -0.001 | -0.018, 0.017 | 1 | |
|  | 570 | 1200 | -0.002 | -0.020, 0.016 | 1 | |
|  | 855 | 1200 | -0.001 | -0.019, 0.017 | 1 | |
| AN | 570 | 855 | 0.001 | -0.012, 0.014 | 1 | |
|  | 570 | 1200 | 9.22E-05 | -0.013, 0.013 | 1 | |
|  | 855 | 1200 | -0.001 | -0.014, 0.012 | 1 | |
| **Synchrony** | | | | | | |
| DMN | 570 | 855 | -0.009 | -0.067, 0.049 | 1 | |
|  | 570 | 1200 | -0.014 | -0.072, 0.044 | 1 | |
|  | 855 | 1200 | -0.005 | -0.063, 0.053 | 1 | |
| CEN | 570 | 855 | -0.003 | -0.059, 0.054 | 1 | |
|  | 570 | 1200 | -0.014 | -0.071, 0.042 | 1 | |
|  | 855 | 1200 | -0.011 | -0.068, 0.045 | 1 | |
| SAL | 570 | 855 | -0.004 | -0.062, 0.053 | 1 | |
|  | 570 | 1200 | -0.011 | -0.069, 0.046 | 1 | |
|  | 855 | 1200 | -0.007 | -0.064, 0.050 | 1 | |
| SMN | 570 | 855 | -0.007 | -0.065, 0.050 | 1 | |
|  | 570 | 1200 | -0.016 | -0.073, 0.042 | 1 | |
|  | 855 | 1200 | -0.008 | -0.066, 0.049 | 1 | |
| VN | 570 | 855 | 0.004 | -0.057, 0.065 | 1 | |
|  | 570 | 1200 | -7.38E-05 | -0.061, 0.061 | 1 | |
|  | 855 | 1200 | -0.004 | -0.065, 0.057 | 1 | |
| AN | 570 | 855 | -0.004 | -0.061, 0.053 | 1 | |
|  | 570 | 1200 | -0.008 | -0.065, 0.049 | 1 | |
|  | 855 | 1200 | -0.004 | -0.061, 0.053 | 1 | |
| MD = mean difference; CI = confidence interval; DMN = default mode network; CEN = central executive network; SAL = salience network; SMN = sensorimotor network; VN = visual network; AN = auditory network. | | | | | | |

**Supplemental References**

1 Ponce-Alvarez, A. *et al.* Resting-state temporal synchronization networks emerge from connectivity topology and heterogeneity. *PLoS Comput Biol* **11**, e1004100, doi:10.1371/journal.pcbi.1004100 (2015).

2 Lachaux, J. P., Rodriguez, E., Martinerie, J. & Varela, F. J. Measuring phase synchrony in brain signals. *Hum Brain Mapp* **8**, 194-208 (1999).

3 Gu, S. *et al.* Emergence of system roles in normative neurodevelopment. *Proc Natl Acad Sci U S A* **112**, 13681-13686, doi:10.1073/pnas.1502829112 (2015).

4 Smith, S. M. *et al.* Resting-state fMRI in the Human Connectome Project. *Neuroimage* **80**, 144-168, doi:10.1016/j.neuroimage.2013.05.039 (2013).
